# Nurses' Role in Caring for Women Experiencing Intimate Partner Violence in the Sri Lankan Context

**DOI:** 10.5402/2012/486273

**Published:** 2012-07-16

**Authors:** Sepali Guruge

**Affiliations:** Daphne Cockwell School of Nursing, Ryerson University, 350 Victoria Street, Toronto, ON, Canada M5B 2K3

## Abstract

Intimate partner violence has short- and long-term physical and mental health consequences. As the largest healthcare workforce globally, nurses are well positioned to care for abused women. However, their role in this regard has not been researched in some countries. This paper is based on a qualitative study that explored how Sri Lankan nurses perceive their role in caring for women who have experienced partner violence. Interviews with 30 nurses who worked in diverse clinical and geographical settings in Sri Lanka revealed that nurses' role involved: identifying abuse, taking care of patients' physical needs, attending to their safety, providing support and advice, and making referrals. Barriers to providing care included lack of knowledge; heavy workload; language barriers; threats to personal safety; nurses' status within the healthcare hierarchy; and lack of communication and collaboration between various stakeholder groups within the healthcare system. Nurses also identified a lack of appropriate services and support within hospitals and in the community. The findings reveal an urgent need for the healthcare system to respond to nurses' educational and training needs and help them function autonomously within multidisciplinary teams when caring for abused women. The findings also point to a need to address institutional barriers including the lack of appropriate services for abused women.

## 1. Introduction

Intimate partner violence (IPV) is defined as the threat of and/or actual physical, sexual, psychological, or verbal abuse and control by a current or former spouse or nonmarital partner [1]. IPV is a global epidemic, and it has been linked to a range of short- and long-term physical and mental health consequences [1]. Consequently, women dealing with IPV or its aftermath are more likely than women with no history of abuse to use health services including emergency departments, outpatient clinics, and in-patient units [2]. Each of these visits affords an opportunity for healthcare professionals to address women's experiences of IPV and minimize negative health consequences [2]. As the largest healthcare workforce globally, nurses are more likely to interact with women experiencing IPV than any other healthcare professionals. Researchers have long been interested in nurses' role in caring for women dealing with IPV, but the topic has not been investigated in some countries. This gap is a major impediment to improving the response of local health sectors to this serious health concern. This paper presents the findings from the first study to explore nurses' role in caring for women who have experienced IPV in the Sri Lankan context. The findings may have implications for other low-income countries.

## 2. Background

### 2.1. The Roles of Nurses and Other Healthcare Professionals in Caring for Victims of IPV

Previous research has demonstrated that healthcare professionals have various beliefs that influence their response to IPV, such as: family is sacred, abuse is rare or is usually a one-time occurrence, abuse is a private matter, a certain amount of physical violence is to be expected within families, and reported violence needs to be verified [1–7]. These beliefs, in addition to their own experiences of violence and trauma and their concerns about offending patients by intruding into private matters, often prevent healthcare professionals from providing appropriate care and support to women experiencing IPV. Previous research has demonstrated that IPV is usually not an isolated event and that it tends to increase in frequency and severity, so lack of an appropriate response from healthcare professionals contributes to continued morbidity and mortality risks for women [2, 8, 9].

### 2.2. IPV and the Sri Lankan Context

Few studies have focused on IPV in Sri Lanka; the available data come from diverse sources such as newspaper reports, police, and hospital records, or small studies conducted by nongovernmental organizations, sociologists, lawyers, and healthcare professionals. A number of these small studies have reported a high prevalence of IPV, ranging from 54–83% [10–13]. Research has demonstrated that women's responses to IPV varied: seek immediate safety, cry, hit back, hide, go to their mothers' houses, or consider suicide, but few informed the police [13]. The women often obtained primary support from their mothers, while other family members also helped by giving advice, offering shelter, giving food, and assaulting the abuser [13]. Many lawyers in Sri Lanka have advocated for laws that would provide a more effective societal response to IPV. As a result, legislation was passed in 2005 to allow women to obtain protective orders against their husbands. Physicians in Sri Lanka have also begun to focus more on IPV, but attempts by other healthcare professionals to address IPV are not well documented. In particular, no previous research has examined the roles of nurses in Sri Lanka caring for women who have experienced IPV.

## 3. The Project

### 3.1. Objectives

This paper is based on a study that was conducted to explore how Sri Lankan nurses perceive their roles in caring for women experiencing IPV, and the research questions addressed in this paper are as follows: *(1) What are nurses' perceptions of their role in caring for women experiencing IPV in the Sri Lankan context; (2) What are the barriers nurses face in providing appropriate care to women living with IPV in the Sri Lankan context?*

### 3.2. Methods

A qualitative interpretive descriptive design, which helps to move beyond description to explore meanings and explanation that may have implications for practice [14] was used in this study. 

Participants were recruited from October–December 2007 through announcements and advertisements at the nursing school at the Open University of Sri Lanka (OUSL) and through word of mouth. Each participant was given a small honorarium to defray the costs of participating.

In total, 30 registered nurses (most of whom had enrolled in the Diploma to Degree program at OUSL) participated in the study. They were diverse with regard to ethnicity (Sinhalese, Tamil, and Muslim), age (range = 22–50 years), religion (Buddhist, Hindu, Christian, and Islam), nursing training (government-funded nursing school training or private hospital-based training), length of work experience (range = 1–15 years), work setting (from public health to surgical nursing), and where they lived in Sri Lanka.

The author conducted the interviews in Sinhala (the majority language in Sri Lanka), English, or both English and Sinhala using an open-ended, unstructured interview format to ensure sufficient flexibility to capture the diverse perspectives of participants. Some of the questions posed included the following: *What is your understanding of IPV in the Sri Lankan context? What has been your experience caring for women living with IPV? What roles do nurses play in caring for women living with IPV? What barriers have you faced in providing care to women living with IPV? What barriers do nurses face in providing care to women living with IPV?* Interviews averaged one hour and were audiotaped. 

### 3.3. Ethical Considerations

Prior to the start of the study, ethics approval was obtained from Ryerson University and OUSL. All potential participants were informed, verbally and via written consent form (in their preferred language, Sinhala, Tamil, or English), about their right to refuse to participate or to answer any questions, or to terminate participation at any time. Interviews were conducted in a quiet room at the nursing school at OUSL or another place convenient for participants, and at a time convenient for both the participant and the author.

### 3.4. Data Analysis

A research assistant translated interviews conducted in Sinhala into English and transcribed these and the interviews conducted in English; the author randomly checked transcriptions for accuracy. Data analysis involved thematic analysis. All transcripts were read line by line to develop a preliminary coding scheme, which was then used as a guide to code the remaining interviews. The coding scheme was modified as new interviews were coded. Codes were organized using NVivo software. The subcategories already developed were compared with the new codes to determine commonalities and variations and to develop categories. 

### 3.5. Rigour/Trustworthiness

The strategies employed to ensure rigour/trustworthiness [15] included member checks, peer debriefing, an audit trail, reflexive journaling, and gathering diverse perspectives. 

## 4. Results

The findings are organized into two main areas: the roles of nurses in caring for women who have experienced IPV, and the barriers they face in doing so.

### 4.1. The Role of Nurses in Caring for Women Who Have Experienced IPV

Interviews revealed that nurses played the following roles when caring for women who have experienced IPV: identifying abuse, taking care of patients' physical health needs, attending to their safety, making referrals, and providing support and advice. 

#### 4.1.1. Identifying Abuse

Almost all participants referred to the importance of identifying IPV, but they had various opinions about what nurses currently do. Some said nurses could not do anything unless the woman disclosed abuse; in other words, the onus was on the patient:

* If a patient reveals that she has such a problem only then do we interview (I # 21). *

Some said that victims would not disclose IPV even after being asked about it. Others said that a disclosure of abuse depended on the relationship between the patient and the nurse:

*They would be open with you only if you have good communication and are able to talk to them well. I would talk to the woman but I would not head straight to the topic. I would ask her about herself and maybe share a few of my personal details… not a lot. By then sometimes they, on their own, talk about IPV. Once we come to the topic we can talk at length about it. If we can communicate in a way they feel is genuine they won't try to hide things. Instead we refrain from pursuing further if the patient is unwilling to talk (I # 1).*

Overall, most said they made an extra effort to develop a trusting relationship only if they suspected abuse. None suggested screening all patients for abuse, even though most said they believed the rates of IPV in the country were high. 

#### 4.1.2. Taking Care of the Physical Health Needs of Patients

Most patients were admitted to the hospitals for physical injuries or physical health problems, so it was not surprising that most nurses said it was important to take care of the physical health needs of patients:

*We treat her for her injuries but we are unable to do anything further if she doesn't come out with it. If they disclose abuse we are very supportive (I # 4).*

*We ask them what happened and treat the injuries. Other than that at the outpatient department, we decide whether to admit them or to send home. If the woman refuses to go home because of abuse, we try to intervene (I # 11).*

As shown by these two excerpts, some participants referred to their attempts to move beyond the physical aspects of health. However, these attempts appear to have been made only for patients who had disclosed abuse.

#### 4.1.3. Attending to Women's Safety

Nurses stressed that addressing the safety of women (and their children) was a key aspect of their role, and referred to calling the authorities:

*I called and told the husband that I will report him to the Women and Children's Bureau if he didn't admit her to the hospital immediately (I # 6).*

*We had to hand over the child to the police. If such a dangerous thing happens we have to report it to the police (I # 29).*

Only a few women's shelters are available in Sri Lanka, and the nurses were aware that getting physically away from the abuser was not an option for most of their female patients. In a few cases, participants mentioned asking victims to go to their mother's or sister's home to get away from the abuser. 

#### 4.1.4. Making Referrals

Participants described referring women to a number of places or people, including counselling services, the police, the Judicial Medical Officer, and/or temporary shelters:

*We let them know that there are organizations that help and we direct them to those organizations (I # 3).*

*We try to find out what kind of help she needs… if she needs legal help or if she wants to send her husband for counselling (I # 11).*

Participants complained about the lack of counselling options in Sri Lanka, and the lack of response from the healthcare system regarding this concern. Most participants referred to one main agency that does provide counselling, but this agency is located in Colombo, Sri Lanka's capital city, making it difficult for many victims to access.

#### 4.1.5. Providing Support

Participants said they provided various kinds of support to abused women: psychological, informational, instrumental, and financial.

*We try to relieve their mental stress by talking to them (I # 27).*

*We visit slums and the husband is sexually abusing her, and sometimes they have about 10 children and we provide information about family planning (I # 19).*

*If she wants financial independence we try to help her find a job. The problem is this kind of intervention is not easy and we cannot make sure she gets every meal (I # 5).*

Additionally, some participants spoke about spending their own money to buy food and/or clothes for the abused women. These gestures were extraordinary, given that some of the nurses were in difficult financial situations or had families of their own to support. 

Participants also described providing other types of support, including getting the husband to sign forms to enable the woman to have necessary health procedures, ensuring that steps needed for legal intervention were appropriately completed, and raising awareness about abuse among relatives and in the community:

*Because nurses can be called upon to testify during legal procedures, we can keep safe her clothes and such. We can also raise awareness amongst her relatives that this is illegal and that they must help her during this difficult time (I # 3).*

#### 4.1.6. Providing Advice

Participants reported giving advice to the woman about leaving the abuser, taking legal action, or going to the authorities. However, as revealed by the following excerpts, the most frequent kind of advice was about living peacefully/tolerating/trying harder:

*I would advise her to try and understand her husband… I would tell her that despite everything this is her husband and she should listen to him. If she is at fault I would try and correct her… I can refer them to certain places. I would not recommend her to get a divorce because there are other men who are waiting to prey on a woman like that. As you get older you need the security and companionship of a man (I # 10).*

*If she has children we try to advise them to somehow work things out because a family should remain as a family. We try to soothe her mind a little and tell her that she needs to remain calm and try to be happy and keep the family together (I # 20).*

A few participants clearly stated that women should not ever be abused and/or should not have to suffer to remain in a marriage, but most felt that women should remain with their husbands. Most of their rationales for this opinion were related to the safety and/or welfare of the children and/or women, and to their belief in the importance of preserving the family. Given the limited services and options for abused women in Sri Lanka, many participants felt that advising them to leave their husband would be pointless. Further, given the general attitudes in Sri Lanka about divorced or separated women, many nurses were concerned that women living alone could potentially face unsafe situations for themselves and their children. 

Some participants also referred to advising the husband about the health, social, and economic consequences of his actions toward his wife:

*We have small sessions in our clinics and we tell the husbands that they have to look after their wives, make sure to feed the children, and give her money to manage the daily expenses (I # 26).*

*In front of the wife we would get the husband and ask him not to abuse her because she just had a baby as well (I # 9).*

However, nurses were not certain of the effectiveness of their actions, due to their limited ability to follow up with their patients following their discharge from the hospitals or clinics.

### 4.2. The Barriers Faced by Nurses in Providing Care to Women Who Have Experienced IPV

The participants identified a number of barriers to supporting abused women appropriately: lack of knowledge and skills; heavy workload and lack of time; language barriers; threats to personal safety; nurses' status within the healthcare hierarchy/system; and lack of communication and collaboration between various stakeholder groups within the healthcare system. These are described next.

#### 4.2.1. Lack of Knowledge and Skills

Nurses identified the lack of knowledge and skills as a key barrier to providing suitable care for women living with IPV, particularly a lack of awareness about IPV, its health consequences, and how nurses can intervene:

*Other staff might ask us why we are asking about abuse. They may think we are just interested in the story. They do not see this [IPV] as a health issue (I # 1).*

*Mental health is not looked at. Social issues are not looked at. This is a huge gap. In front of everybody she is asked if she were beaten and the woman is already in a bad state. Sometimes they look at the woman as if it's her fault that she got beaten up. Because the foundation itself that is laid in nursing is not good enough, and this situation is maintained by the management, the hospital, and the healthcare system (I # 17).*

*We don't have any special lectures or in-service education on IPV, so we gain knowledge through experience. So junior nurses without much experience might not be able to successfully handle these cases and might just focus on the physical aspect. If we are given training we could intervene successfully instead of just making referrals (I # 13).*

These gaps in knowledge also meant that nurses tended to focus on the physical health of patients. Participants saw the lack of protocol about addressing non-physical health issues as contributing to their limited clinical skills in caring for women living with IPV. Many participants said they thought they could improve in their role if they were given appropriate training.

#### 4.2.2. Heavy Workload and Lack of Time

All participants raised concerns about their workload and the limited time available for building a trusting relationship that would enable them to identify IPV or create a safe space for the patients to disclose abuse:

*We are often short-staffed. They should increase the field staff especially the percentage of female staff. There are very few public health staff and we don't have time for follow up (I # 24).*

*We don't get time to do counselling because the workload is stifling and the patient-nurse ratio is very high (I # 11).*

Nurses across clinical and geographical settings identified overwhelming workload and lack of time as key barriers to providing appropriate care. The exceptions were the few participants who worked in private hospitals who did not identify these as major concerns.

#### 4.2.3. Language Barriers

Although participants did not frequently refer to language barrier, some participants noted that language could be problematic, primarily for Tamil and Tamil-speaking Muslim patients. The onus appeared to be on the patient to understand Sinhala or bring an interpreter. However, some nurses tried various strategies to help patients who do not speak Sinhala:

*There is a girl in the minor staff who is fluent in Tamil so we get her help or sometimes I speak to the Tamil doctors but there is no formal process (I # 10).*

*I am slightly conversant in Tamil. If a Tamil-speaking patient comes, I go out of my way to help the patient if it is necessary. But it does not happen always with other nurses (I # 27).*

While language barriers could also affect Sinhalese women living in primarily Tamil-speaking areas of the country, the issue was raised only by participants working in Sinhala-speaking regions.

#### 4.2.4. Threats to Nurses' Safety

Personal safety was another concern that participants raised in the context of working with women living with IPV. This was especially an issue for those working in rural settings or villages and/or visiting patients' homes:

*It poses a threat to the lives of the nurses as well. So we have to be very prudent because we travel and live by ourselves sometimes and our safety becomes a concern (I # 2).*

*We try to talk to them but other than that we don't get involved, because of threats to our safety. There are all sorts of complications if one does that. If we get into trouble who will protect us? (I # 18).*

Some participants said that their nursing uniform acted as a sign of authority and provided a measure of safety, but that in small towns and villages it is easy to find out who lives where. Female nurses who lived alone said that they too might be at risk of harm from their patients' abusive husbands, but male participants did not voice such concerns. Overall, hospital administrators were perceived as being unreceptive to the vulnerability of the nurses.

#### 4.2.5. The Status of Nurses within the Healthcare Hierarchy

Participants said that the nurses were at the bottom of the healthcare hierarchy, and they were, therefore, unable to make independent decisions about how to help women living with IPV. Nurses had to follow the chain of command regardless of the situation, and the participants said that it was difficult to get everyone's buy-in for the kind of care the women deserved:

*There is a huge gap between the nurses and the nursing sisters. The matron steps out and all other nurses turn to dust. This attitude is a huge barrier (I # 17).*

*A consultant would want another doctor's recommendation to take a patient and the recommendation of the sister is not enough (I # 6).*

*We don't do independent decisions. We always consult doctors or link with legal officials. We take the instructions of our superiors. Nurses are limited to trolley care (I # 23).*

These comments exemplify how nurses have limited authority to act independently. They seem to be controlled not only by the medical profession, but also from within the nursing profession, where a clear hierarchy is maintained from nurses to nursing sisters to Matrons.

#### 4.2.6. Lack of Communication and Collaboration between Various Stakeholder Groups within the Healthcare System

Some participants discussed the lack of communication and lack of equal sharing of information across various disciplines within the healthcare system. With a limited followup system, each group often ended up working in “silos.” For example,

*Nurses and midwives working in the community don't communicate well about these issues…There is no connection between the hospital and the community either (I # 5).*

Some participants felt that management and hospitals had no interest in improving patient care, working conditions for nurses, or communication between multidisciplinary team members, even though they felt that management was fully aware of how these issues affected patients and nurses.

Participants also identified several key deficiencies within the current Sri Lankan healthcare system. They said that no system had been developed to care for abused women in hospitals, and no formal system of followup care was available. They also reported that on the few occasions when psychological or other counsellors are available in the hospital, the wards provided no space for private conversations with patients. They also alluded to a lack of organized care within the community:

*The other problem with our healthcare system is that most of our nurses are in hospitals… we should have more nurses working in the community (I # 10).*

Many participants reported that the medical profession had prevented attempts to develop a community health nursing system. Thus, they felt that changes could only occur after a major overhaul of the healthcare system. 

## 5. Study Limitations

The study findings would have been enriched by including the perspectives of women about the care they had received (or would like to have received). Despite this limitation, the findings of this study have important implications for nurses, nursing, and healthcare systems.

## 6. Discussion

The findings reveal that while most participants believed that the rates of IPV are high in Sri Lanka and that it is important to identify IPV, they placed the onus on victims to disclose abuse and did not routinely ask patients about IPV. These findings are similar to those reported in other countries (see, e.g., [3, 16–20]). Together, these findings also confirm that even when IPV is “suspected” or identified, nurses often do not intervene effectively (e.g., they tend to focus only on physical health).

The findings of the current study demonstrate that nurses in Sri Lanka need adequate knowledge and skills to respond appropriately to the unique needs of women living with IPV. Studies conducted in various healthcare settings and countries have identified lack of education and training as key barriers to the response of healthcare professionals to IPV (see, e.g., [6, 21–29]). These studies have also identified important aspects to include in curricula about IPV: health consequences of IPV, cultural and societal taboos and myths about IPV, asking about IPV, responding to a patient's disclosure of IPV, conducting danger/safety assessments, making referrals to appropriate resources within hospitals and in the community, child protection, and documentation. 

Since the 1990s, researchers have reported that nurses, in particular, receive inadequate education and training about IPV (see, e.g., [16, 28–34]). More than 20 years later, nursing curricula in many countries remain unchanged in this regard. In Sri Lanka, nursing curricula are still based on the medical model and are driven by the medical profession; they have yet to incorporate appropriate content about IPV. Nurses need to be trained and supported to function autonomously within multidisciplinary teams within the health care system, to provide effective care for women who have experienced IPV. 

This study identified various institutional barriers that affect how nurses care for women experiencing IPV, including a lack of support from various levels of nursing staff as well as from other healthcare professionals, management, hospitals, and the healthcare system. Some nurses referred to collegial collaboration with doctors who supported nurses' decisions to ask about IPV and provide care, support, and referrals to women who have experienced IPV, but most participants referred to the lack of such support. They also identified other institutional barriers, such as lack of time, space, and privacy in healthcare settings as impediments to providing appropriate care for abused women. Based on similar research in other countries, Häggblom et al. [17] and Lynch [35] also reported a lack of support for nurses from management and hospitals. According to Häggblom et al. study participants, support from health authorities for women living with IPV was “almost absent” [17]. In the context of Sri Lanka, De Silva and Rolls [36] wrote, “poor hospital management allows doctors to admit too many patients, resulting in chaotic and overcrowded work environments with unsustainable resources” (p. 33). The constrained environment and the strict professional hierarchy under which nurses work have a negative impact on their ability to care for abused women. Nurses need to address their own experiences of oppression before they can help others. This is an area requiring serious attention by all stakeholders involved. Furthermore, hospital management need to address nurses' safety concerns. 

Research has suggested that a more comprehensive and systems-based approach may be necessary to affect the frequency and quality of assessing for/asking about IPV by healthcare professionals [37, 38]. This kind of approach may include implementation of institutional policies, protocols and prompts; on-site resources such as victim advocates or social workers; practice changes recommended by the accrediting body; and referral and support for staff who may be experiencing IPV [32, 35, 39]. 

Although IPV has become a major priority for health worldwide, in many countries the top levels of healthcare systems exhibit a lack of or limited interest in addressing this topic. In the context of global health, a recent WHO report on IPV [1] noted, “public health approaches to violence can make a difference; however, this potential is far from being realized in many countries” (p. viii). In Sri Lanka, a stronger public and community health response is vital to mitigate the significant health problems faced by women, their children, and families as a result of IPV. Efforts to help women who have experienced IPV must go beyond caring for individual patients, and aim to change the cultural, political, and social contexts that produce and maintain IPV.

## 7. Conclusions

The high prevalence of IPV in Sri Lanka highlights the urgent need for the healthcare system to respond to the educational and training needs of nurses in diploma and degree programs, and the need for continuing education in various practice settings. To ensure that this change in curricula leads to actual practice changes, all other healthcare professionals must also receive education and training to collaborate with nurses in addressing IPV in a range of clinical settings. Practice protocols, guidelines, policies, and prompts must be developed and implemented along with actual services and programs to which women living with IPV can be referred for further social, legal, and other supports. This can be achieved only with responsive management within a supportive health care system. Finally, all healthcare professionals must work together to advocate for services such as counselling and shelters to meet the needs of the abused women, and to change the cultural, political, and social contexts that produce and maintain high rates of IPV.
